# Relationships between food consumption and living arrangements among university students in four European countries - A cross-sectional study

**DOI:** 10.1186/1475-2891-11-28

**Published:** 2012-04-24

**Authors:** Walid El Ansari, Christiane Stock, Rafael T Mikolajczyk

**Affiliations:** 1 Faculty of Sport, Health & Social Care, University of Gloucestershire, Gloucester, United Kingdom; 2 Unit for Health Promotion Research, Institute of Public Health, University of Southern Denmark, Esbjerg, Denmark; 3 Dept. of Public Health Medicine, School of Public Health University of Bielefeld, Bielefeld, Germany; 4 Department of Clinical Epidemiology, Bremen Institute for Prevention Research and Social Medicine, Achterstraße 30, Bremen, D-28359, Germany

**Keywords:** University students, Dietary intake, Fruits and vegetables, Food consumption, CNSHS

## Abstract

**Background:**

The transition of young people from school to university has many health implications. Food choice at the university can differ because of childhood food consumption patterns, sex and the living arrangements. Food consumption may change especially if students are living away from home. We aimed to assess food consumption patterns among university students from four European countries and how they differ by their living arrangements.

**Methods:**

We analysed data from a cross-country survey assessing health and health behaviours of students. The sample comprised a total of 2402 first year undergraduate students from one university in each of the countries of Germany, Denmark, Poland and Bulgaria. Food consumption was assessed by means of a food frequency questionnaire with 9 food groups (indicators).

**Results:**

Students’ food consumption patterns differed across the countries. Frequent consumption of unhealthy items was common. Bulgarian students reported most often frequent consumption of sweets and cakes and snacks (e.g. chips and fast food). Polish students reported the least frequent consumption of vegetables and a low consumption of fruits. Across all countries except Bulgaria, men reported substantially more often frequent consumption of snacks than women. Students living at parental home consumed more fruit, vegetables, and meat than those who resided outside of their family home in all studied countries. There was more variation with regard to cakes and salads with more frequent consumption of cakes among Bulgarian female students and Danish male students and more frequent consumption of salads among Danish female students not living at parental home, compared to students from other countries.

**Conclusions:**

Nutrition habits of university students differed across countries and by sex. Students living at parental home displayed more healthy nutrition habits, with some exceptions.

## Background

The transition of young people from school to university has many health implications. It is a time of increased responsibility for food choices and practices [1]. A key concern is the food consumption patterns and associated nutritional risks specific to college students [2,3]. The nutritional knowledge of university students [4,5], and their diets (food consumption patterns) have received global attention [1,4,6,7].

Changes in living arrangements that some college students encounter influence their lifestyle factors e.g. food choices [8]. University students’ diets feature some undesirable practices, especially for those living away from the family home [9]. In Greece, students living away from the family home made some positive changes (e.g. decrease in whole-fat dairy products, white bread and margarine), but they simultaneously decreased their consumption of fresh fruit, cooked and raw vegetables, oily fish, and increased their sugar and fast food intake [10]. Polish female students living away from their parents had significantly less percentage of energy provided by total fat and higher percentage of energy from carbohydrate than students who reside with their parents [11].

Surveys on eating behaviour of young people and adolescents have been conducted in many European countries (e.g. [12-15]). However, most studies examined single countries and some have focused only on one sex e.g. [16]. Cross-national comparisons of diet quality have also been undertaken (mostly only two countries), but focussed on the general populations (e.g. [17]) or adolescents (e.g. [18]) rather than university students. Similarly, cross-national research of the food habits of university students mostly compared only two countries [19,20]. Exceptions include the European Prospective Investigation into Cancer and Nutrition that examined the general populations (10 European countries) [21]; and the International Health Behaviour Study that examined the food choice behaviours in university students (23 countries) [22]. Besides these, less research has compared the cross-national variations in dietary habits of *university* students across *several* countries, employing more than a single student population and hence a wide variation in diet, using large samples, and measuring the frequency of a wide range of dietary intakes.

Therefore, the present study surveyed a large sample of students from two western European (Denmark, Germany) and two eastern European countries (Bulgaria, Poland). The aims were: 1) to describe and examine variations by gender and university site (country) of food consumption habits of students, and 2) to examine the relationship between food consumption and students’ living arrangements during university term (whether at home with their parents, or residing outside of their family home).

## Methods

### Participants

The dataset used in this analysis is part of the Cross National Student Health Survey (CNSHS), a general health survey among student populations conducted in several European countries [23]. In the current analysis four sites were included, which conducted the study in 2005: University of Bielefeld, Germany (DE); University of Southern Denmark (DK); Catholic University of Lublin, Poland (PL); and Sofia University, Bulgaria (BG). The study applied a self-administered multi-theme health survey, including questions on multiple health behaviours. The original questionnaire was developed in German and translated into Polish, Bulgarian and Danish by two independent translators. The two versions were compared and their disagreements resolved in discussion with native speakers involved in the research team. The questionnaire was distributed to first-year students during regular classes in the final part of selected courses and collected immediately after being completed by the students. No incentives were provided. The response rates (based on numbers of returned questionnaires) were >95% (Bulgaria, Poland), 92% (Denmark), and varied (60%–100%) for Germany depending on the surveyed groups (85% on average, lower response rates in large lecture rooms than in smaller seminars). The study included 2651 participants. Sex was missing for 60 students and an additional 189 had missing responses in some questions resulting in a final sample size of 2402.

### Measures

A food frequency questionnaire (9 indicator variables) assessed consumption patterns of sweets; cakes/cookies; snacks and fast/canned food; fresh fruits; raw and cooked vegetables and salads; and meat and fish (see Table 1). The introductory question “How often do you eat the following foods?” asked participants about the frequency of their usual consumption of each food item separately (rated on a 5 point scale: several times a day, daily, several times a week, 1–4 times a month, and never). These 5 categories were collapsed into two categories: high frequency (several times a day/daily) and low frequency (several times a week/1–4 times a month/never). In the case of three items (cakes, snacks and fish), high frequency was defined as several times a day/daily/several times a week versus low frequency 1–4 times a month/never. The instrument was based on pre-existing food frequency questionnaires, adapted for the study and used in previous publications [24]. Both the face and content validity of the instrument were ascertained by grounding the questionnaire on literature review. No formal test of validity was performed, but the questionnaire was very similar to other food frequency questionnaires that had been validated, e.g. [25,26]. The question: “Where do you live during university/college term time?” assessed students’ living arrangements during university terms, with four closed options and one open end response (alone; with my partner; with my parents; with room-mates; other). This variable was recoded into two options: at parental home versus all other options.

**Table 1:** Sociodemographic characteristics of the study population by country

	**Germany**		**Denmark**		**Poland**		**Bulgaria**		
	**N**	**%**	**N**	**%**	**N**	**%**	**N**	**%**	p-value**
Sex									
Female	444	57.7%	267	48.7%	408	71.3%	479	68.3%	<0.001
Male	326	42.3%	281	51.3%	164	28.7%	222	31.7%	
Age [years]									
<20	14	1.8%	29	5.3%	132	22.9%	387	55.2%	<0.001
20-23	602	78.7%	369	67.3%	439	76.2%	300	42.8%	
>23	149	19.5%	150	27.4%	5	.9%	14	2.0%	
Living with parents*									
No	486	64.4%	491	90.9%	395	70.2%	406	58.8%	<0.001
Yes	269	35.6%	49	9.1%	168	29.8%	285	41.2%	

* living at parental home during university during term/semester.

** chi-square test by country.

### Informed consent and ethical permission

Participation in the study was voluntary and anonymous. Students were informed that by completing the questionnaire they were providing their informed consent to participate. They were also informed that they could terminate the participation at any point while filling out the questionnaire. The permission to conduct the study was granted by the participating institutions: Bielefeld University in Bielefeld (Germany), the Catholic University of Lublin, Lublin (Poland), the University of Sofia in Sofia (Bulgaria) and the University of Southern Denmark (Denmark).

### Statistical analysis

The analysis was conducted using SPSS 17 (SPSS Inc., Chicago, IL, USA), with statistical significance level set at p <0.05. First, the sociodemographic characteristics of the sample were presented by country and tested for differences using chi-square test. Second, the consumption of food items by country, by sex and by both was described based on the dichotomised values. Bivariate comparisons were tested by chi-square test. When country and sex were considered simultaneously, logistic regression with main effects of country and sex and their interaction was conducted to obtain significance tests. When the interaction was not significant, it was removed and the model was re-estimated using main effects only. Third, the effects of not living at parental home on the consumption of the food groups were assessed in logistic regression models. Each sex-country combination was recoded into a new variable yielding 8 distinct groups. The interaction between this new variable and living at parental home versus other living arrangements indicated whether the effects were homogenous or varied across countries or by sex. When interaction term was significant, stratified analysis was carried out to present the effects across the sex/country combinations.

## Results

### Characteristics of the sample

The proportion of females was double or more the males in the two Eastern European countries and nearly equal in the two Western European countries (Table 1). About 70-80% of students in the German, Danish and Polish samples were aged 20 – 23 years, while about half (55%) in Bulgaria and one fifth (23%) in Poland were <20 years. Students aged >23 years represented < 1% in Poland but were about a quarter of the Danish sample . In Poland, Bulgaria and Germany, 25%-50% of participants lived at home with their parents during their studies; in Denmark this proportion was much lower. Across the four countries, generally more males than females lived at parental home (data not shown).

### Food consumption by country and sex

Across the four countries under examination, Bulgarian students most often reported frequent consumption of the ‘less healthy’ items (sweets, cakes, snacks and fast foods) (Table 2). Conversely, Bulgarian students also most often reported frequent consumption of some ‘healthy’ items including salads (59%) and vegetables (32%). As regards meat, the percentage of students reporting frequent consumption was relatively high in all countries (ranging from 44%-53%). High frequency of fish consumption was most commonly reported by Bulgarian students.

**Table 2:** Consumption of food items by country and by sex

	**Country**					**Sex**		
	Germany	Denmark	Poland	Bulgaria	p-value**	Female	Male	p- value***
	N=							
Sweets*	33.0	15.2	28.7	52.8	<0.001	39.0	25.2	<0.001
Cakes^†^	27.7	18.9	60.6	72.2	<0.001	49.1	38.7	<0.001
Snacks^†^	28.2	14.7	25.8	60.9	<0.001	32.6	35.2	0.17
Fast food^†^	33.6	19.6	10.6	77.1	<0.001	32.7	45.3	<0.001
Fruits*	41.4	41.7	35.0	49.6	<0.001	48.9	31.4	<0.001
Salads*	32.5	34.6	27.2	58.7	<0.001	44.0	30.4	<0.001
Vegetables*	25.9	19.3	15.2	31.6	<0.001	24.4	22.3	0.22
Meat*	44.2	52.7	46.3	47.4	0.022	38.5	61.5	0.001
Fish ^†^	26.6	34.9	29.2	38.1	<0.001	28.7	37.3	<0.001

* Percentage reporting high consumption (several times a day/daily).

^†^ Percentage reporting consumption at least several times per week (several times a day/daily/several times per week).

** chi-square test by country.

*** chi-square test by sex.

Overall, more women than men reported frequent consumption of sweets, cakes, fruits and salads, while more men reported frequent consumption of fast food, meat and fish. Both sexes reported frequent consumption of vegetables at a comparable level.

When country and sex were considered at the same time (Table 3), most of the differences observed in bivariate analyses persisted. At the same time most of the country and sex effects were independent and significant interactions existed only for salads and fish consumption. For salads consumption particularly strong differences by sex existed in Germany and Denmark, in contrast to Poland and Bulgaria where consumption frequency in both sexes was similar. With respect to fish consumption, there was nearly no sex difference in Denmark, while in other countries males reported consistently more often frequent consumption.

**Table 3:** Consumption of food items by country and sex

	**Germany**		**Denmark**		**Poland**		**Bulgaria**		p-value**		
**Food Item**	**Female**	**Male**	**Female**	**Male**	**Female**	**Male**	**Female**	**Male**	**Country**	**Sex**	**Country x sex**
	N = 394	N = 302	N = 262	N = 278	N = 351	N = 138	N = 449	N = 205			
Sweets*	38.2	26.3	21.5	9.3	30.6	22.2	56.5	45.7	<0.001	<0.001	n.a.
Cakes^†^	28.5	26.9	19.2	18.6	63.3	54.8	73.1	70.4	<0.001	0.11	n.a.
Snacks^†^	23.0	34.7	12.7	16.5	22.5	32.7	60.8	61.5	<0.001	<0.001	n.a.
Fast food^†^	25.1	44.5	11.2	27.6	7.5	18.8	72.4	87.7	<0.001	<0.001	n.a.
Fruits*	47.8	32.1	55.4	28.6	39.5	23.8	54.1	39.4	<0.001	<0.001	n.a.
Salads*	37.5	25.4	46.4	23.3	28.0	23.9	61.8	51.6	<0.001	<0.001	0.018
Vegetables*	26.8	24.3	22.6	16.1	15.2	14.4	31.1	32.7	<0.001	0.25	n.a.
Meat*	32.0	60.9	46.1	59.1	41.3	58.5	37.8	67.4	<0.001	<0.001	n.a.
Fish ^†^	21.8	32.9	36.5	33.3	27.0	34.8	31.9	50.7	0.21	0.002	0.005

* Percentage reporting high consumption (several times a day/daily).

^†^ Percentage reporting consumption at least several times per week (several times a day/daily/several times per week).

** p-values from logistic regression including main effects for country and sex and the interaction between them both.

n.a. – when interaction term was not significant it was removed and significance of main effects was studied.

### Comparison of dietary patterns of students having ‘other’ living arrangements with those living at parental home

In the case of most food groups, the effect of living away from parental home was homogenous across all country/sex combinations (Table 4). While the effect was not significant for the consumption of sweets, snacks, fast food or fish, the consumption of fruits, vegetables and meat was less frequent among students living away from parental home (Table 4). For cakes and salads there was some heterogeneity across the country/sex combinations (Table 5). In both cases, the consumption was lower among those living away from parental home for most country/sex combinations as indicated by odds ratios below 1. The exception was a higher consumption of cakes among students living away of parental home among Bulgarian females and Danish males. In the case of salads, in general the consumption was lower among those living away from parental home with the exception of Danish females who had higher consumption of salads when they were not living at parental home.

**Table 4:** Odds ratios of frequent consumption for students living at ‘other’ accommodation compared to students living at parental home (for food groups with homogenous effects by country/sex)

**Food Group**	**‘Other’ accomodation versus parental home**	**p-value**
	**OR (95% CI)**^**a**^	
Sweets*	0.98 (0.81-1.19)	0.82
Snacks^†^	1.08 (0.88-1.31)	0.47
Fast food^†^	1.16 (0.93-1.44)	0.2
Fruits*	0.75 (0.63-0.90)	0.002
Vegetables*	0.46 (0.38-0.57)	<0.001
Meat*	0.62 (0.52-0.75)	<0.001
Fish^†^	0.83 (0.68-1.01)	0.06

^a^ Odds ratio of high versus low consumption among those living at ‘other’ accommodation compared to students living at parental home, adjusted for all country/sex combinations, separate models for each food group.

* Frequent consumption defined as: several times a day/daily.

^†^ Frequent consumption defined as: several times a day/daily/several times per week.

**Table 5:** Odds ratios of frequent consumption for students living at ‘other’ accommodation compared to students living at parental home (for food groups with heterogeneous effects by country/sex)

	**Females**				**Males**			
	**Germany**	**Denmark**	**Poland**	**Bulgaria**	**Germany**	**Denmark**	**Poland**	**Bulgaria**
Food group	OR (95% CI)^a^	OR (95% CI)^a^	OR (95% CI)^a^	OR (95% CI)^a^	OR (95% CI)^a^	OR (95% CI)^a^	OR (95% CI)^a^	OR (95% CI)^a^
Cakes^†^	0.46 (0.30-0.73)	0.36 (0.13-1.05)	0.66 (0.41-1.07)	1.34 (0.87-2.04)	0.62 (0.37-1.02)	1.31 (0.48-3.57)	0.58 (0.30-1.15)	0.51 (0.28-0.93)
Salads*	0.79 (0.52-1.21)	1.53 (0.54-4.32)	0.47 (0.29-0.76)	0.35 (0.23-0.54)	0.70 (0.42-1.16)	0.35 (0.17-0.75)	0.69 (0.33-1.46)	0.20 (0.11-0.36)

^a^ odds ratio (95% CI) for high frequency versus low frequency consumption among those living at ‘other’ accommodation compared to students living at parental home, analysis stratified by sex/country, each food group was analysed separately; numbers below 1 indicated that consumption among those not living at home was less common; similar numbers indicate similarities across the countries and by sex.

^†^ Frequent consumption defined as: several times a day/daily/several times per week.

* Frequent consumption defined as: several times a day/daily.

## Discussion

This article reports food consumption habits in young people attending universities in four countries. University students are usually better educated and younger than a population-based sample, and comprise a suitable sample for examining food consumption habits, as the variability of ill health and education is minimal.

Regarding the first aim of the study, students’ nutrition showed some unfavourable practices across all studied sites. E.g. less than 50% of students reported frequent consumption of fruits. High consumption of foods that are “away” from the Mediterranean diet [where standard dessert is fresh fruit with sweets consumed only on special occasions [27]] was significantly associated with 10-year coronary risk of >10% for 40% of adults [28]. Other studies among students confirmed a low intake of fruits and vegetables [29-31]. However, students’ food consumption patterns differed also across the sites. For instance, Bulgarian female and male students reported most often a frequent consumption of sweets and cakes. Bulgarian students also had highest frequencies of snacks, including chips (40%), and fast food (52%). This could indicate a need for specific public health action at this site.

Women’s food consumption differed significantly from men’s. For snacks, across all 4 countries, men had reported more often a frequent consumption of snacks than women, supporting similar findings from France that male university students had a higher mean number of daily snacks than women [32]. For fruits, Lee [30] found that female students had better nutritional habits than men (were more likely to report eating fruit/vegetables), providing support that women behaved ‘healthier’ than men in terms of fruit consumption [32]. Earlier reports also found that female university students ate more fruits than males [33]. However a point is that although females might consume more fruits than men, such consumption levels could still fall short of recommended daily recommendations [29]. We are in agreement with these authors, where across our sample, women exhibited more fruit, salads and vegetable consumption than men. Regardless of country, more males regularly consumed meat than females, in agreement with Turkish males who reported significantly more meat serving per day compared to female adolescents [29]. Our findings also support results of Swedish university students where females had healthier habits related to nutrition [34].

Regarding the second aim of the study, across the four countries, 59% to 91% of students were not living at home with their parents during their studies, similar to France where at least 65% of university students lived away from the family home during the week [33]. Our findings suggested that students living at home with their parents consumed more fruits and cooked/raw vegetables than those who resided outside of their family home. This parallels Papadaki’s [10] conclusions that students living away from the family home decreased their weekly consumption of fresh fruit and cooked/raw vegetables. This might suggest parents’ potential influence on their children’s diets, in agreement with studies which found associations between intakes of parents and their adolescent children for fruit, vegetables and dairy foods [35,36]. Indeed parents can possibly inspire their children’s food intake positively through role modeling and the food environment they provide at home [37,38]. Furthermore, home accessibility to fruits and vegetables was found to increase preference for these foods in a 6-month follow-up study [39].

Students living away from the family home might develop more unfavourable eating habits than those living at the family home. This might be due to the fact that those living at home do not have to pay for food and therefore do not suffer from financial limitations in this respect. In addition, meals containing vegetables and other healthy food items might be prepared for them and thus more healthy food is available to them. As college students leave home and adjust to independent living, good dietary habits decline [40]. The living situation is further compounded, where studies have reported that students living on campus reported significantly less frequent food preparation [41]. Frequency of preparing food was related to more healthful food choices in terms of lower intakes of fat and fried foods and higher intakes of fruits and vegetables [42]. These factors affect students when they move to a different city within their own country [43], or translocate to attend university in countries other than their own with new eating patterns and food choices in their new environment [9,44]. This might be particularly relevant for the consumption of fast foods. In our sample, living away from parental home was not associated with higher consumption of fast food, snacks or sweets. Fast foods consumption, a frequently used indicator of unhealthy eating [45], might mirror the degree of the shift away from traditional cooking towards meals that are made outside the home [46]. Indeed the consumption of nutritious foods (e.g. fruit, vegetables) is inversely related to the frequency of visits to fast food outlets [47,48]. Although we found a lower consumption in healthy food items like fruits and vegetable in students who have moved out from parental home this was not accompanied by a significant increase in fast food, snacks or sweet consumption in our samples.

Our study has several limitations. Data were self-reported (no validation undertaken) and cross-sectional (does not infer causal relationships). Food consumption was estimated with 9 food frequency questions. Although similar (or shorter) questionnaires have been alleged to underestimate fruit and vegetable or high-fat foods intake when compared to multiple 24-hour dietary recalls [49], for fruit and vegetable, they produced estimates similar to those produced by other brief food frequency surveys [50]. We did not undertake formal tests of validity on the questionnaire but it was very similar to other food frequency questionnaires: e.g. Osler & Heitmann’s [25] for which validity was demonstrated that it correctly quantified food intakes when compared with diet history interview; and to Roddam et al’s [26] who reported that the short food group questionnaire functioned reasonably well for the assessment of dietary nutrients. In Bulgaria, the frequency of consumption for all studied food items was higher than in other countries, suggesting a specific tendency of responses. While the translation very strongly focussed on the equivalence of meaning, there are some cultural aspects of translation which cannot be overcome. Possibly this was here the case, but we were not able to assess this issue formally. Another limitation is that we were not able to further differentiate living conditions with respect to opportunities for food preparation. Residence halls are not common in the studied countries, but still the conditions for food preparation can differ across different living arrangements. In fact, we were only able to make a meaningful separation between those living at parental home and those not, while we were not able to compare different living arrangements among those not living at home. We did not assess whether the students were eating at the university’s refectories or were preparing their own food. We also did not assess if differences between those living at parental home or not were possibly resulting from pre-existing choices in food consumption rather than introduced by change in living arrangements. Furthermore, we examined only one university per country, differences between countries could be in fact just differences between particular universities. Theoretically, students with particular nutrition patterns could be also more prone to choose universities which require that they leave parental home.

## Conclusions

Food consumption patterns differed across the studied countries, with females typically making more healthy choices. Differences between students living at parental home and not were relatively homogenous across the countries, i.e. despite differences in background patterns of food consumption leaving parental home is associated with specific patterns of food consumption. If those changes are really introduced by leaving parental home should be subject of further longitudinal studies. In such a case, specific health education programs and other interventions could be proposed to address unfavourable changes and support those which likely improve health.

## Competing interests

The authors declare no competing interests.

## Authors’ contributions

WEA and RTM developed the research question. RTM conducted the analysis. WEA drafted the manuscript and CS and RTM provided comments. All authors accepted the final version.

## References

[B1] Colic Baric I Satalic Z Lukesic Z Nutritive value of meals, dietary habits and nutritive status in Croatian university students according to gender Int J Food Sci Nutr 2003 54 473 484 10.1080/09637480310001622332 14522693

[B2] Gores SE Addressing nutritional issues in the college-aged client: strategies for the nurse practitioner J Am Acad Nurse Pract 2008 20 5 10 10.1111/j.1745-7599.2007.00273.x 18184159

[B3] Krinke U Brown J Adult nutrition Nutrition Through the Life Cycle 2002 Wadsworth / Thomson Learning, Belmont, CA 383 407

[B4] Broccia F Lantini T Luciani A Carcassi AM Nutrition knowledge of Sardinian and Corsican university students Ann Ig 2008 20 49 55 18478676

[B5] Kolodinsky J Harvey-Berino JR Berlin L Johnson RK Reynolds TW Knowledge of current dietary guidelines and food choice by college students: better eaters have higher knowledge of dietary guidance J Am Diet Assoc 2007 107 1409 1413 10.1016/j.jada.2007.05.016 17659910

[B6] Farghaly NF Ghazali BM Al-Wabel HM Sadek AA Abbag FI Life style and nutrition and their impact on health of Saudi school students in Abha, Southwestern region of Saudi Arabia Saudi Med J 2007 28 415 421 17334472

[B7] Brunt A Rhee Y Zhong L Differences in dietary patterns among college students according to body mass index J Am Coll Health 2008 56 629 634 10.3200/JACH.56.6.629-634 18477517

[B8] Brevard PB Ricketts CD Residence of college students affects dietary intake, physical activity, and serum lipid levels J Am Diet Assoc 1996 96 35 38 10.1016/S0002-8223(96)00011-9 8537567

[B9] Kremmyda LS Papadaki A Hondros G Kapsokefalou M Scott JA Differentiating between the effect of rapid dietary acculturation and the effect of living away from home for the first time, on the diets of Greek students studying in Glasgow Appetite 2008 50 455 463 10.1016/j.appet.2007.09.014 17997195

[B10] Papadaki A Hondros G A Scott J Kapsokefalou M Eating habits of university students living at, or away from home in Greece Appetite 2007 49 169 176 10.1016/j.appet.2007.01.008 17368642

[B11] Jaworowska A Bazylak G Residental factors affecting nutrient intake and nutritional status of female pharmacy students in Bydgoszcz Rocz Panstw Zakl Hig 2007 58 245 251 17711118

[B12] Hagman U Bruce A Persson LA Samuelson G Sjolin S Food habits and nutrient intake in childhood in relation to health and socio-economic conditions. A Swedish Multicentre Study 1980–81 Acta Paediatr Scand Suppl 1986 328 1 56 3471046

[B13] Anderson AS Macintyre S West P Dietary patterns among adolescents in the west of Scotland Br J Nutr 1994 71 111 122 10.1079/BJN19940116 8312235

[B14] Spyckerelle Y Herbeth B Deschamps JP Dietary behaviour of an adolescent French male population J Hum Nutr Diet 1992 5 161 168 10.1111/j.1365-277X.1992.tb00151.x

[B15] Bull NL Dietary habits, food consumption, and nutrient intake during adolescence J Adolesc Health 1992 13 384 388 10.1016/1054-139X(92)90034-9 1390792

[B16] Irazusta A Gil S Ruiz F Gondra J Jauregi A Irazusta J Gil J Exercise, physical fitness, and dietary habits of first-year female nursing students Biol Res Nurs 2006 7 175 186 10.1177/1099800405282728 16552945

[B17] Kim S Haines PS Siega-Riz AM Popkin BM The Diet Quality Index-International (DQI-I) provides an effective tool for cross-national comparison of diet quality as illustrated by China and the United States J Nutr 2003 133 3476 3484 14608061 10.1093/jn/133.11.3476

[B18] Chen MY James K Wang EK Comparison of health-promoting behavior between Taiwanese and American adolescents: a cross-sectional questionnaire survey Int J Nurs Stud 2007 44 59 69 10.1016/j.ijnurstu.2005.11.015 16386741

[B19] Sakamaki R Amamoto R Mochida Y Shinfuku N Toyama K A comparative study of food habits and body shape perception of university students in Japan and Korea Nutr J 2005 4 31 10.1186/1475-2891-4-31 16255785 PMC1298329

[B20] Miere D Filip L Indrei LL Soriano JM Molto JC Manes J Nutritional assessment of the students from two European university centers Rev Med Chir Soc Med Nat Iasi 2007 111 270 275 17595880

[B21] Key TJ Appleby PN Rosell MS Health effects of vegetarian and vegan diets Proc Nutr Soc 2006 65 35 41 10.1079/PNS2005481 16441942

[B22] Wardle J Haase AM Steptoe A Nillapun M Jonwutiwes K Bellisle F Gender differences in food choice: the contribution of health beliefs and dieting Ann Behav Med 2004 27 107 116 10.1207/s15324796abm2702_5 15053018

[B23] El Ansari W Maxwell AE Mikolajczyk RT Stock C Naydenova V Kramer A Promoting public health: benefits and challenges of a Europeanwide research consortium on student health Cent Eur J Public Health 2007 15 58 65 17645218 10.21101/cejph.a3418

[B24] Mikolajczyk RT El Ansari W Maxwell AE Food consumption frequency and perceived stress and depressive symptoms among students in three European countries Nutr J 2009 8 31 10.1186/1475-2891-8-31 19604384 PMC2716364

[B25] Osler M Heitmann BL The validity of a short food frequency questionnaire and its ability to measure changes in food intake: a longitudinal study Int J Epidemiol 1996 25 1023 1029 10.1093/ije/25.5.1023 8921490

[B26] Roddam AW Spencer E Banks E Beral V Reeves G Appleby P Barnes I Whiteman DC Key TJ Reproducibility of a short semi-quantitative food group questionnaire and its performance in estimating nutrient intake compared with a 7-day diet diary in the Million Women Study Public Health Nutr 2005 8 201 213 15877913 10.1079/phn2004676

[B27] Kafatos A Verhagen H Moschandreas J Apostolaki I Van Westerop JJ Mediterranean diet of Crete: foods and nutrient content J Am Diet Assoc 2000 100 1487 1493 10.1016/S0002-8223(00)00416-8 11138441

[B28] Panagiotakos D Sitara M Pitsavos C Stefanadis C Estimating the 10-year risk of cardiovascular disease and its economic consequences, by the level of adherence to the Mediterranean diet: the ATTICA study J Med Food 2007 10 239 243 10.1089/jmf.2006.251 17651058

[B29] Bas M Altan T Dincer D Aran E Kaya HG Yuksek O Determination of dietary habits as a risk factor of cardiovascular heart disease in Turkish adolescents Eur J Nutr 2005 44 174 182 10.1007/s00394-004-0509-8 15309435

[B30] Lee RL Loke AJ Health-promoting behaviors and psychosocial well-being of university students in Hong Kong Public Health Nurs 2005 22 209 220 10.1111/j.0737-1209.2005.220304.x 15982194

[B31] Skemiene L Ustinaviciene R Piesine L Radisauskas R Peculiarities of medical students’ nutrition Medicina (Kaunas) 2007 43 145 152 17329950

[B32] Monneuse MO Bellisle F Koppert G Eating habits, food and health related attitudes and beliefs reported by French students Eur J Clin Nutr 1997 51 46 53 10.1038/sj.ejcn.1600361 9023467

[B33] Schweyer FX Le Corre N L’alimentation au quotidien chez les e’tudiants Pre’venir 1994 26 87 9222893881

[B34] von Bothmer MI Fridlund B Gender differences in health habits and in motivation for a healthy lifestyle among Swedish university students Nurs Health Sci 2005 7 107 118 10.1111/j.1442-2018.2005.00227.x 15877687

[B35] Young EM Fors SW Factors related to the eating habits of students in grades 9–12 J Sch Health 2001 71 483 488 10.1111/j.1746-1561.2001.tb07285.x 11816396

[B36] Hanson NI Neumark-Sztainer D Eisenberg ME Story M Wall M Associations between parental report of the home food environment and adolescent intakes of fruits, vegetables and dairy foods Public Health Nutr 2005 8 77 85 10.1079/PHN2005661 15705248

[B37] Hill L Casswell S Maskill C Jones S Wyllie A Fruit and vegetables as adolescent food choices in New Zealand Heal Promot Int 1998 13 55 65 10.1093/heapro/13.1.55

[B38] Baranowski T Cullen KW Baranowski J Psychosocial correlates of dietary intake: advancing dietary intervention Annu Rev Nutr 1999 19 17 40 10.1146/annurev.nutr.19.1.17 10448515

[B39] Bere E Klepp KI Changes in accessibility and preferences predict children’s future fruit and vegetable intake Int J Behav Nutr Phys Act 2005 2 15 10.1186/1479-5868-2-15 16216124 PMC1262749

[B40] Harris KM Gordon-Larsen P Chantala K Udry JR Longitudinal trends in race/ethnic disparities in leading health indicators from adolescence to young adulthood Arch Pediatr Adolesc Med 2006 160 74 81 10.1001/archpedi.160.1.74 16389215

[B41] Larson NI Perry CL Story M Neumark-Sztainer D Food preparation by young adults is associated with better diet quality J Am Diet Assoc 2006 106 2001 2007 10.1016/j.jada.2006.09.008 17126631

[B42] Larson NI Story M Eisenberg ME Neumark-Sztainer D Food preparation and purchasing roles among adolescents: associations with sociodemographic characteristics and diet quality J Am Diet Assoc 2006 106 211 218 10.1016/j.jada.2005.10.029 16442868

[B43] Arvaniti F Panagiotakos DB Pitsavos C Zampelas A Stefanadis C Dietary habits in a Greek sample of men and women: the ATTICA study Cent Eur J Public Health 2006 14 74 77 16830608 10.21101/cejph.a3374

[B44] Pan YL Dixon Z Himburg S Huffman F Asian students change their eating patterns after living in the United States J Am Diet Assoc 1999 99 54 57 10.1016/S0002-8223(99)00016-4 9917732

[B45] Schmidt M Affenito SG Striegel-Moore R Khoury PR Barton B Crawford P Kronsberg S Schreiber G Obarzanek E Daniels S Fast-food intake and diet quality in black and white girls: the National Heart, Lung, and Blood Institute Growth and Health Study Arch Pediatr Adolesc Med 2005 159 626 631 10.1001/archpedi.159.7.626 15996994

[B46] Kant AK Graubard BI Eating out in America, 1987–2000: trends and nutritional correlates Prev Med 2004 38 243 249 10.1016/j.ypmed.2003.10.004 14715218

[B47] Bowman SA Vinyard BT Fast food consumption of U.S. adults: impact on energy and nutrient intakes and overweight status J Am Coll Nutr 2004 23 163 168 15047683 10.1080/07315724.2004.10719357

[B48] Paeratakul S Ferdinand DP Champagne CM Ryan DH Bray GA Fast-food consumption among US adults and children: dietary and nutrient intake profile J Am Diet Assoc 2003 103 1332 1338 10.1016/S0002-8223(03)01086-1 14520253

[B49] Lowry R Galuska DA Fulton JE Wechsler H Kann L Collins JL Physical activity, food choice, and weight management goals and practices among US college students Am J Prev Med 2000 18 18 27 10808979 10.1016/s0749-3797(99)00107-5

[B50] Field AE Colditz GA Fox MK Byers T Serdula M Bosch RJ Peterson KE Comparison of 4 questionnaires for assessment of fruit and vegetable intake Am J Public Health 1998 88 1216 1218 10.2105/AJPH.88.8.1216 9702152 PMC1508294

